# 

**Published:** 1894-02-17

**Authors:** 


PRICE TWOPENCE.
No. 386, Vol. XV.—Feb. 17th, 1894.
[Registered at the General Post Office as a Newspaper for transmission in the United Kingdom and Abroad.]

WITH EXTRA NURSING SUPPLEMENT.
Per Annum, 8s. 6d., post free.
Monthly Parts, 6d.; post free, 8d.
Ditto per Annum, 8s., post free.

### THE HOSPITAL

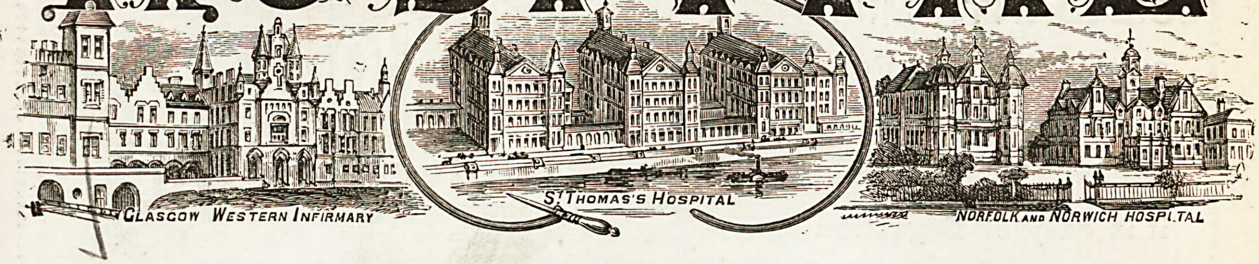

No. 386. Vol. XV. WITH EXTRA NURSING SUPPLEMENT. Saturday, February 17th, 1894.

